# Development of a membrane-based Gi-CASE biosensor assay for profiling compounds at cannabinoid receptors

**DOI:** 10.3389/fphar.2023.1158091

**Published:** 2023-08-11

**Authors:** Morgan Scott-Dennis, Fikri A. Rafani, Yicheng Yi, Themiya Perera, Clare R. Harwood, Wolfgang Guba, Arne C. Rufer, Uwe Grether, Dmitry B. Veprintsev, David A. Sykes

**Affiliations:** ^1^ Division of Physiology, Pharmacology and Neuroscience, School of Life Sciences, University of Nottingham, Nottingham, United Kingdom; ^2^ Centre of Membrane Proteins and Receptors (COMPARE), University of Nottingham, Midlands, United Kingdom; ^3^ Roche Pharma Research and Early Development, Roche Innovation Center Basel, F Hoffmann-La Roche Ltd, Basel, Switzerland; ^4^ Z7 Biotech Limited, London, United Kingdom

**Keywords:** G-protein signaling, Gα_i_ signaling pathway, cannabinoid receptors (CBRs), inverse agonism, BRET—bioluminescence resonance energy transfer, GPCR signalling assay, Gα_i_ signalling assay

## Abstract

**Introduction:** The cannabinoid receptor (CBR) subtypes 1 (CB_1_R) and 2 (CB_2_R) are key components of the endocannabinoid system (ECS), playing a central role in the control of peripheral pain, inflammation and the immune response, with further roles in the endocrine regulation of food intake and energy balance. So far, few medicines targeting these receptors have reached the clinic, suggesting that a better understanding of the receptor signalling properties of existing tool compounds and clinical candidates may open the door to the development of more effective and safer treatments. Both CB_1_R and CB_2_R are Gα_i_ protein-coupled receptors but detecting Gα_i_ protein signalling activity reliably and reproducibly is challenging. This is due to the inherent variability in live cell-based assays and restrictions around the use of radioactive [^35^S]-GTPγS, a favoured technology for developing higher-throughput membrane-based Gα_i_ protein activity assays.

**Methods:** Here, we describe the development of a membrane-based Gα_i_ signalling system, produced from membrane preparations of HEK293TR cells, stably overexpressing CB_1_R or CB_2_R, and components of the Gα_i_-CASE biosensor. This BRET-based system allows direct detection of Gα_i_ signalling in both cells and membranes by monitoring bioluminescence resonance energy transfer (BRET) between the α and the βγ subunits. Cells and membranes were subject to increasing concentrations of reference cannabinoid compounds, with 10 μM furimazine added to generate RET signals, which were detected on a PHERAstar FSX plate reader, then processed using MARS software and analysed in GraphPad PRISM 9.2.

**Results:** In membranes expressing the Gi-CASE biosensor, the cannabinoid ligands profiled were found to show agonist and inverse agonist activity. Agonist activity elicited a decrease in the BRET signal, indicative of receptor activation and G protein dissociation. Inverse agonist activity caused an increase in BRET signal, indicative of receptor inactivation, and the accumulation of inactive G protein. Our membrane-based Gi-CASE NanoBRET system successfully characterised the potency (pEC_50_) and efficacy (E_max_) of CBR agonists and inverse agonists in a 384-well screening format. Values obtained were in-line with whole-cell Gi-CASE assays and consistent with literature values obtained in the GTPγS screening format.

**Discussion:** This novel, membrane-based Gα_i_ protein activation assay is applicable to other Gα_i_-coupled GPCRs, including orphan receptors, allowing real-time higher-throughput measurements of receptor activation.

## Introduction

CB_1_R and CB_2_R are class A, rhodopsin-like G protein-coupled receptors (GPCRs) and signal primarily through Gα_i_ proteins, which inhibit downstream cellular signalling processes (Mackie, 2008). Cannabinoid receptors are reportedly involved in the control of endogenous pain (Pertwee, 2001), neuronal excitation and development (Durieux et al., 2022), memory formation (Scienza-Martin et al., 2022), and modulate inflammation (Jiang et al., 2022) and immune responses (Llorente-Ovejero et al., 2022). Recent evidence highlights ECS signalling in thyroid, adrenal and gonadal function (Meah et al., 2022), building on its well-known endocrine role in food intake and energy balance (Di Marzo and Matias, 2005). Therefore, drugs targeting CB_1_R and CB_2_R are often evaluated for their anti-nociceptive, anti-anxiolytic, anti-inflammatory properties and in endocrine-related disorders such as obesity (Modaresi and Talachian, 2022). Few drugs targeting CB_1_R and CB_2_R are currently marketed, due to often serious CB_1_R-related neurological and other unwanted side effects, which limit their clinical utility. Therefore, the development of agents with an improved side-effect profile could transform the current cannabinoid drug landscape. To increase a drug’s therapeutic potential, we can improve its selectivity for a particular target, or alternatively, we can attempt to generate a unique receptor activation profile, one which permits the recruitment of therapeutic effector proteins at the expense of those causing side effects (so-called ligand bias). Currently, the detection of more selective or biased ligands for GPCRs is hampered by a lack of high throughput screening (HTS) techniques, which can reveal subtle differences in receptor efficacy or bias towards a particular effector protein following receptor activation.

Almost all GPCRs signal via coupling to heterotrimeric G proteins, consisting of an α, β and γ subunit. A single receptor can couple to multiple G proteins by interacting with the Gα subunit, which could fall into one of four families, namely, Gα_s_, Gα_i_, Gα_q_, and Gα_12_. Of the 134 GPCRs with approved drugs, the Gα_i_ protein is most often targeted, thus highlighting the therapeutic importance of cyclic adenosine monophosphate (cAMP) regulation by current therapeutic agents (Sriram and Insel, 2018). Gα_i_ proteins inhibit the activity of adenylyl cyclase (AC), and thus the production of cAMP. Monitoring Gα protein coupling via AC in a HTS format requires prior stimulation of cAMP production, usually with a specific activator of AC such as forskolin. This artificial manipulation of the cAMP signal complicates the assay, increasing the potential for biological variability in terms of cell responsiveness. For example, the protean CB_2_R-selective ligand AM1241 can behave as a cAMP agonist or antagonist depending on the concentration of forskolin used to activate AC (Yao et al., 2006). The quantification of cAMP is often carried out by indirect detection methods involving the use of antibodies. Such indirect measures can often lead to higher levels of assay-to-assay variability, which is partly a consequence of the multiple sample preparation steps involved and the reliance on a standard curve.

To overcome these challenges, the [^35^S]-GTPγS binding assay can be used to directly detect Gα_i_ protein activation and inactivation (Strange, 2010). This assay constitutes a functional measure of the interaction of the receptor and the G protein. The advantages of this assay in comparison to other second messenger detection assays are such that the assays are relatively simple and use bulk-frozen membranes. Membranes are a highly desirable screening format, providing improved day-to-day consistency and convenience over cell-based assays. This assay format also has a lower degree of receptor reserve, meaning that the fraction of receptors required to produce a maximal system response (E_max_) is much greater compared to other functional assays (Sykes et al., 2009; Buchwald, 2019). Therefore, this system is ideal for differentiating full and partial agonists and understanding overall receptor selectivity.

However, the [^35^S]-GTPγS binding assay has a relatively low signal-to-background ratio. This is especially true for Gα_s_-coupled receptors due to the lower abundance of the Gα_s_ protein in cells (Harrison and Traynor, 2003). Another major drawback of this format is the need for designated working spaces, the provision of protective equipment and the requirement of regular radiation monitoring. In addition, [^35^S]-GTPγS has a relatively short half-life (87.8 days), making the assays expensive and inconvenient to run on a routine basis.

As an alternative to the [^35^S]-GTPγS binding assay, we have used a BRET-based biosensor with the potential to be converted into a membrane-based detection system and capable of ranking compounds based on both efficacy (E_max_) and potency (pEC_50_). Whole-cell based systems using a BRET-based G protein dissociation biosensor were first developed by the Bouvier lab (Gales et al., 2005), with similar biosensors developed by the Roth lab (Olsen et al., 2020), who identified optimal labelling sites in individual G protein subunits. More recently, these sensors have been further refined by the Schulte lab, who produced the first G protein–based tricistronic activity sensors (G-CASE biosensors), which allow the expression of all three G protein subunits from a single plasmid (Schihada et al., 2021), thus simplifying their use. These whole-cell systems are perfect for detecting the activity of agonists and inverse agonist but are currently a low throughput technique, which is generally utilised in a 96-well format and requires an initial washing step to remove cell growth media in exchange for assay buffer. To address this, we have developed a membrane-based 384-well system that is not only more convenient and reproducible but has the potential to be further miniaturised.

**FIGURE 1:**
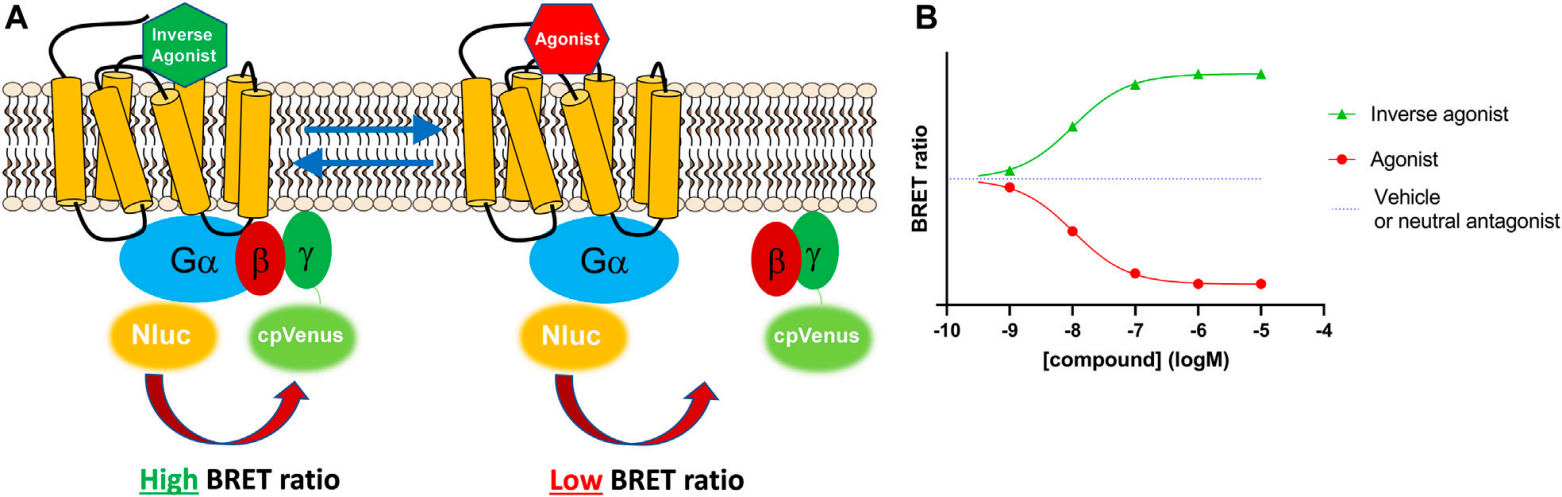
The Gi-CASE biosensor detects G protein activity based on changes in BRET signal. **(A)** Gi-CASE assay signal detection. The Gi-CASE biosensor plasmid genetically incorporates a NanoLuciferase^®^ donor on the Gα subunit and a Venus fluorescent acceptor fluorophore on the *N*-terminus of the Gγ subunit. Agonist-induced G protein activation results in dissociation of the Gα and Gβγ subunits causing a reduction in the BRET signal. Inverse agonist activity causes a reduction in Gα and Gβγ dissociation and promotes reassociation resulting in an increase in the relative BRET signal. **(B)** Simulated Gi-CASE data, shown as a change in (∆) BRET ratio for an inverse agonist (green) and agonist (red) of equal potency.

Here, we describe a membrane-based signalling system created using the Gi-CASE construct. Firstly, we recreated the whole-cell system by overexpressing the G-CASE biosensor for the Gα_i_ protein (Gi-CASE) in cells stably expressing CB_1_Rs and CB_2_Rs. Gi-CASE biosensors genetically incorporate a NanoLuciferase or NanoLuc^®^ donor and Venus fluorescent acceptor protein to the Gα and Gγ subunit, respectively. Agonist-receptor binding causes G protein activation and the dissociation of the Gα and Gβγ subunits, resulting in a loss of the BRET signal. In comparison, inverse agonists increase the BRET signal by binding the receptor and stabilizing its inactive conformation, thereby initiating G protein inactivation below the level of basal activity by stabilising the heterotrimeric complex, eventually preventing Gα_i_ signalling and the accumulation of free Gα_i_ protein. The principle behind the Gi-CASE assay is presented in Figure 1.

The functional activity of the whole-cell systems was tested using two well-characterised agonists, HU-210 (a dual CB_1/2_R agonist and synthetic cannabinoid) and HU-308 (a selective CB_2_R agonist and cannabidiol derivative), and rimonabant and SR-144,528 (selective inverse agonists of CB_1_R and CB_2_R, respectively) (Devane et al., 1992; Hanus et al., 1999; Stern and Lambert, 2007). By comparing agonist and inverse agonist stimulation in both intact cells and membranes expressing CB_1_R and CB_2_R, we could assess the suitability of the membrane-based system as a replacement for whole cells and evaluate its potential as a universal detector of Gα_i_ protein activation or inactivation. In further experiments, we profiled a collection of known CB_1_R- and CB_2_R-specific ligands including the CB_1_R-specific neutral antagonist AM4113 (Sink et al., 2008). This was done to better understand the effects of temperature on individual ligand responses, and to fully test the assay’s ability to correctly classify cannabinoid molecules in terms of their relative efficacy and potency.

## Materials, instruments and software

### Materials

Human embryonic kidney 293TR (HEK293TR or HEK293-TRE_x_™) cells were obtained from ThermoFisher Scientific. T75 and T175 cm^2^ culture flasks were purchased from ThermoFisher Scientific. Dulbecco’s Modified Eagle’s Medium (DMEM) - high glucose media, Dulbecco’s Phosphate Buffered Saline (DPBS), no calcium, no magnesium (D8537) was purchased from Sigma-Aldrich. CellStripper™ was purchased from Corning. Hanks’ Balanced Salt solution (H8264), HEPES (4-(2-hydroxyethyl)-1-piperazineethanesulfonic acid), EDTA (ethylenediamine tetraacetic acid), bovine serum albumin (BSA) heat shock fraction, protease-free, fatty acid-free, essentially globulin free (A7030), poly-D-lysine, tetracycline and Pluronic F127 were purchased from Sigma-Aldrich. The transfection reagent PEI linear, MW 25000, transfection grade (PEI 25K) was obtained from Polysciences, (23966-1). The selection reagents blasticidin, geneticin (G418) and zeocin were obtained from Invitrogen. A bicinchoninic acid (BCA) protein assay kit, used to determine the total protein content of membranes, was obtained from ThermoFisher Scientific. Rimonabant, SR-144,528, HU-308, JTE-907, JWH-133, WIN55212-2, cannabinol and HU-210 were obtained from Tocris Bioscience (Bristol, United Kingdom). CP55,940, AM1241 and AM4113 was obtained from Sigma-Aldrich. The Gi1-CASE encoding plasmid developed by Schihada *et al.*, was obtained from Addgene (id: 168120). All ligands were dissolved in 100% DMSO and stored as aliquots at −20°C until required. Dimethyl sulfoxide (DMSO, 276855) was purchased from Sigma-Aldrich. OptiPlate-384 (White Opaque 384-well Microplate), were purchased from PerkinElmer (Beaconsfield, United Kingdom).

### Instruments and software

The T 10 homogeniser and the associated S 10 N - 10 G dispersing element were obtained from IKA-England Ltd. Beckman Avanti J-251 ultracentrifuge (Beckman Coulter, California United States). BMG PHERAstar FSX plate reader (BMG Labtech, Offenburg, Germany), fitted with a BRET1 plus (535-30LP/475-30BP) module and MARS software were purchased from BMG Labtech (Offenburg, Germany). GraphPad Prism 9.2 (GraphPad Software, San Diego, United States). Microsoft Excel™ XP was purchased from Microsoft (Washington, United States).

## Methods

### Cell culture

The HEK293TR cell line was used for the generation of stable cell lines expressing either the CB_1_R and CB_2_R and the Gi-CASE biosensor. The human CB_1_R (hCB_1_R) and CB_2_R (hCB_2_R) was stably transfected into HEK293TRs, and the resulting mixed population cell lines were then transfected with the Gi-CASE plasmid. CBR cell lines were made using SNAP-tagged hCB_1_R and hCB_2_R cDNAs encoded in pcDNA4™/TO (Invitrogen, tetracycline-inducible vector). The selection of cells expressing the CB_1_R or CB_2_R, and the Tet-On system was achieved through the application of zeocin (20 μg/mL) and blasticidin (5 μg/mL). Gi-CASE biosensors are encoded by a single Gi1-CASE plasmid that genetically incorporates a NanoLuciferase donor fluorophore on the Gα subunit and a Venus fluorescent acceptor fluorophore to the *N*-terminus of the gamma portion of the Gβγ subunit. The plasmid was stably transfected into HEK293TR cells using PEI and a 3:1 PEI:DNA ratio. The selection of cells expressing the Gi-CASE biosensor was achieved through the application of G418 (0.2 mg/mL). Stable HEK293TR cells expressing CB_1_R and CB_2_R and the Gi-CASE biosensor were cultured in Gibco DMEM - high glucose media, containing 10% foetal calf serum, G418 (0.2 mg/mL), zeocin (20 μg/mL) and blasticidin (5 μg/mL), and L-glutamine. Cell lines were maintained in a humid atmosphere at 37°C and 5% CO_2_ and routinely sub-cultured every 3–4 days, using a split ratio of 1:10.

For the production of HEK293TR membranes containing both CBRs and the Gi-CASE biosensor, cells were grown in t175 cm^2^ flasks. To control CB_1_R and CB_2_R-inducible expression, 1 μg/mL tetracycline was added to the culture medium once cells had reached 80%–90% confluency to inhibit repressor protein binding and stimulate expression of the CBRs. Cells were grown for a further 48 h and were harvested using a non-enzymatic cell dissociation agent CellStripper™ before transferring to a 25 mL universal tube. Cells were then pelleted at 1,200 *g* for 3 min, and the supernatant was removed, before storing the pellets at −80°C.

### Membrane preparation

Buffers used in the preparation of cell membranes include buffer A (10 mM HEPES (4-(2-hydroxyethyl)-1-piperazineethanesulfonic acid), 10 mM EDTA (ethylenediamine tetraacetic acid) pH 7.4) and buffer B (10 mM HEPES, 0.1 mM EDTA, pH 7.4). All the components of the membrane preparation were kept at 4°C during the membrane preparation procedure. Membranes were made by adding 20 mL of ice-cold buffer A to the cell pellet. The pellet was homogenized on ice, using an “ultra-turrax” electrical homogenizer (10 bursts for 1 s on setting 6). The cell homogenate was centrifuged in a 30 mL Sterilin universal container (ThermoFisher) at 1,200 *g* for 3 min (Thermo Scientific Heraeus Megafuge 8) and the pellet containing the cell nuclei and heavy organelles was discarded. The supernatant was then centrifuged at 48,000 *g* for 30 min at 4°C using a Beckman Avanti J-251 ultracentrifuge using the JA-25.50 Fixed-Angle Rotor and Beckman 50 mL centrifuge tubes. The supernatant was removed, and the pellet re-suspended in 0.9 mL buffer B. Aliquots were prepared and snap frozen using liquid nitrogen, prior to storage at −80°C. Protein concentration was determined using the BCA assay, using bovine serum albumin (Sigma-Aldrich) as a standard.

### Intact cell Gi-CASE NanoBRET assay

Receptor activation of CB_1_R- and CB_2_R-expressing cells with the Gi-CASE biosensor was achieved as follows: cells were plated in a white, 96-well clear-bottomed (Greiner 655983) previously coated in poly-D-lysine (5 μg/mL in PBS). After 48–72 h incubation with tetracycline to induce CB_1_R and CB_2_R expression, cell culture media was aspirated off and the cells were then washed (100 μL/well) with assay buffer (Hank’s balanced salt solution (HBSS) containing 0.5% BSA, 5 mM HEPES). Assay buffer (90 μL/well) containing the NanoLuciferase substrate furimazine (10 μM) was dispensed into the wells and the plate was incubated at 37°C for 15 min. The assay plate was transferred to the PHERAstar FSX and three BRET cycles were collected every minute, before adding 10 μL of a 10x stock of compound containing 10% DMSO to the assay plate. Dilutions of the synthetic CBR agonists HU-210 and HU-308, and CB_1_R and CB_2_R inverse agonists rimonabant and SR-144,528 were prepared as follows: compounds were initially serially diluted in DMSO, then diluted 1/10 in assay buffer, before the addition of the compounds to the assay plate. The BRET1 plus (535-30LP/475-30BP) module, which reads at the acceptor excitation wavelength of 535-30LP and the donor emission wavelength of 475-30BP, was used to detect fluorescent G protein activity. Ligands were added after obtaining 3 basal readings to ensure that luminescence levels were sufficient, and to allow for an opportunity to adjust the sensitivity (gain function) on the plate reader. The duration of data collection was >30 min at 1-min intervals (40 cycles).

### Membrane-based Gi-CASE NanoBRET assay

The assay buffer used to profile compounds in our novel membrane-based CB_1_R and CB_2_R Gi-CASE system consisted of HBSS containing 0.02% pluronic F127, 0.5% BSA and 5 mM HEPES. The reference compounds HU-210, HU-308, rimonabant, and SR-144,528 were initially serially diluted in DMSO in a 96-well polypropylene plate. 5 μL of the reference compound serial dilution was then added to 45 µL of assay buffer in a 96-well polypropylene plate. 5 μL of the compound in assay buffer containing 10% DMSO was then added to a white 384-well Optiplate™ (PerkinElmer) containing 35 μL of assay buffer. Finally, the CB_1_R and CB_2_R membranes containing the Gi-CASE biosensor were thawed from −80°C and added into assay buffer containing 50 µM furimazine. Membranes (10 μL) were added to the assay plate at a final concentration of 5 µg/well. The total assay volume was 50 µL. The assay plates were read using a BMG PHERAstar FSX reader at 28°C and 37°C. The duration of data collection on the PHERAstar FSX using the BRET1 plus (535-30LP/475-30BP) module, was 60 min at 1-min intervals (60 cycles) in the case of membrane-based experiments.

### Data analysis

The raw data from all experiments were converted to the ratio of the BRET1 plus module (535-30LP/475-30BP) and moved to Microsoft Excel. The data were then transferred to GraphPad PRISM 9.2. A kinetic analysis of the reference compounds over time was completed by plotting a graph of mean BRET ratios normalised to the vehicle control. Characterisation of agonist CBR responses was achieved by selecting the concentration-response data from membranes at fixed time points which produced maximal responses to the ligands under test. For CB_1_R, this was 10 min after agonist addition or 30 min after inverse agonist addition. For CB_2_R, this point was 30 min after agonist addition or 10min after inverse agonist addition.

Data was normalized and expressed as the change in the BRET signal relative to the vehicle response at the chosen time point.

The graphs were plotted from the normalized data using sigmoidal dose-response (variable slope) or four-parameter logistic equation:

$$Y=Bottom+\frac{\left(Top-Bottom\right)}{1+10^{\left(logEC50-X\right)*\mathrm{Hillslope}}}$$ (1)

Where *Bottom* and *Top* are the plateaus of the agonist and inverse agonist curves. LogEC_50_ is the concentration of agonist/inverse agonist that gives a half-maximal effect and the *Hillslope* is the unitless slope factor. *X* is log of the ligand concentration.

Despite the inverse agonism exhibited by rimonabant and SR-144,528, these compounds are considered competitive antagonists. The pA_2_ value indicates the affinity of the antagonistic agent for the receptor. More precisely, the pA_2_ value is the negative logarithm of the concentration of antagonist needed to shift the dose response curve by a factor of 2. For calculation of the pA_2_ value, the following equation is used

$$pA_{2}=\log\left(DR-1\right)–\log\left[B\right]$$ (2)

Where [B] is the concentration of antagonist used and log DR (dose ratio) is the log ratio of the EC_50_ concentration of the agonist HU-210 in the presence and absence of the antagonist.

The correlation between datasets was determined by calculating a Pearson correlation coefficient (presented as the *r* ^2^ coefficient of determination, which shows percentage variation in y which is explained by all the x variables together) in GraphPad PRISM 9.2.

## Results

### Whole-cell CB_1_R and CB_2_R Gi-CASE responses

**FIGURE 2:**
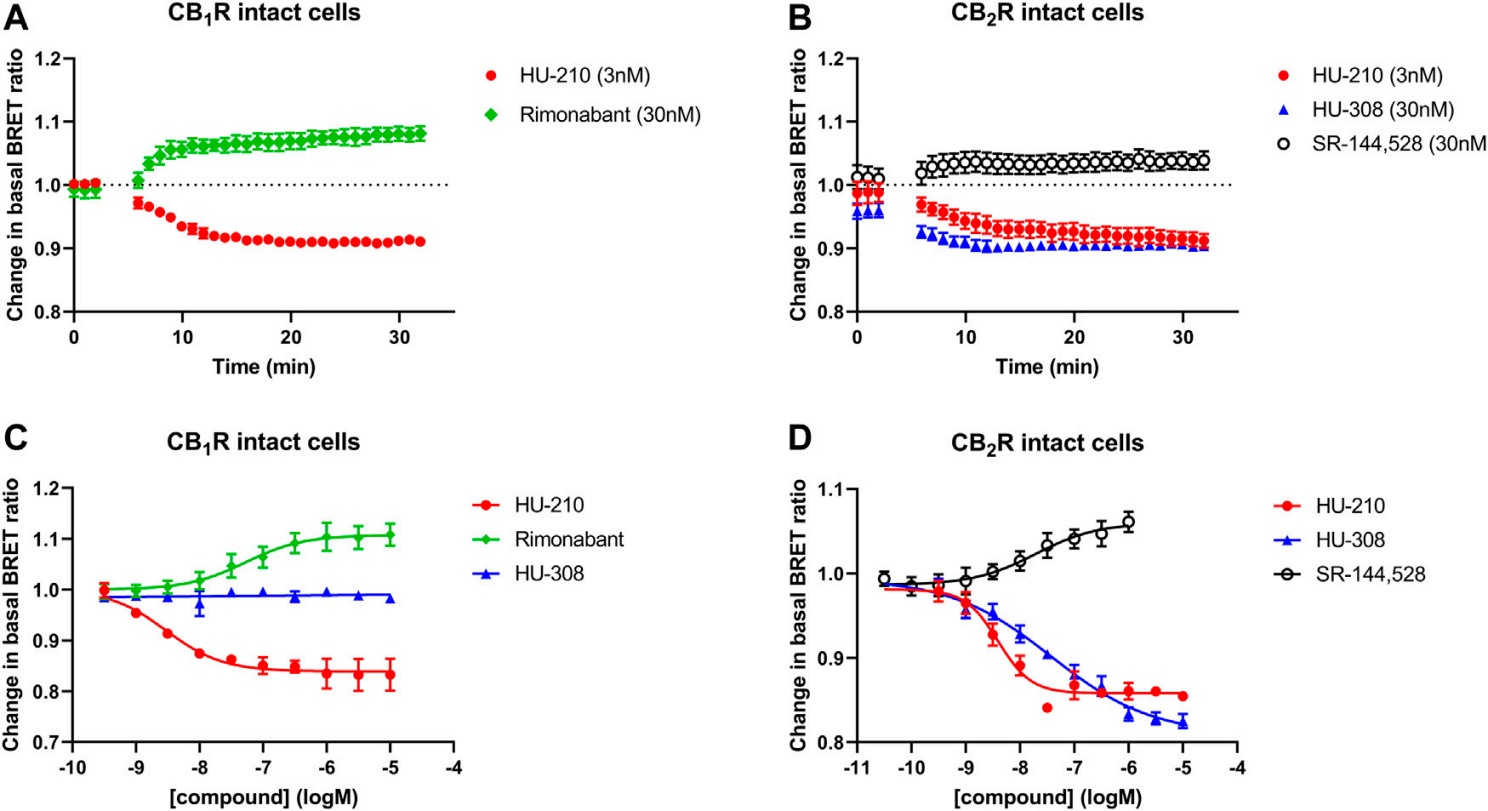
Gi-CASE activation/inhibition time courses and concentration-response curves in CB_1_R and CB_2_R-expressing HEK293TR cells, upon stimulation with reference compounds. Time courses at 37°C of **(A)** CB_1_R- or **(B)** CB_2_R-mediated Gi-CASE activation/inhibition following stimulation with HU-210, HU-308 and rimonabant or SR-144,528 at concentrations around the EC_50_. **(C)** CB_1_R- and **(D)** CB_2_R-mediated Gi-CASE concentration-response curves following stimulation with HU-210, HU-308 and rimonabant or SR-144,528. Data are presented as mean ± S.E.M. from three or more experiments.

Firstly, we characterized the whole-cell BRET response in cells expressing CB_1_R and CB_2_R and the Gi-CASE biosensor, by monitoring the signals at different reference compound concentrations. The response kinetics of the reference agonist (HU-210 and HU-308) and inverse agonist (rimonabant and SR-144,528) ligands at EC_50_ concentrations at both receptors, measured for up to 30 min at 37°C, are shown in Figures 2A, B. Ligands were added after obtaining three basal readings with activation of CB_1_Rs and CB_2_Rs by agonists, resulting in a decrease in the BRET ratio, whilst inverse agonist activity increased the BRET ratio by reducing G protein activation to levels below basal activity. The observed rate of response for the agonists HU-210 and HU-308 appeared to be in part related to the binding kinetics of the ligands, with HU-210 showing the slowest rate of onset. This coincides with its slower rate of dissociation from the CB_2_R compared to the CB_1_R (unpublished data), the dissociation rate being a decisive factor in the rate of receptor occupancy (see Figure 2B). pEC_50_ values of the four reference ligands measured in intact cells at 37°C are summarized in Table 1, and associated response curves at 30 min are presented in Figures 2C, D.

**TABLE 1:** Intact cell-based Gi-CASE signalling assay functional parameters for cannabinoid ligands acting at the human CB_1_ and CB_2_ receptor.

Compound	Intact cell (Assay temperature, 37°C)			
	CB_1_R		CB_2_R	
	pEC_50_	E_max_	pEC_50_	E_max_
HU-210	8.66 ± 0.21 (5)	0.864 ± 0.015	8.60 ± 0.27 (5)	0.838 ± 0.012
HU-308	ND	ND	7.46 ± 0.15 (5)	0.792 ± 0.025
Rimonabant	7.62 ± 0.41 (4)	1.112 ± 0.010	ND	ND
SR-144,528	ND	ND	7.30 ± 0.37 (5)	1.087 ± 0.014

ND, Not determined. Summary table of results from intact cell-based Gi-CASE assays conducted in cannabinoid receptor 1 and 2-expressing HEK293TR cells, in the presence of reference ligands. E_max_ is expressed as change in the basal BRET ratio. Data are averaged (mean ± S.E.M) with the number of observations indicated in parentheses.

### Membrane CB_1_R and CB_2_R Gi-CASE responses

**FIGURE 3:**
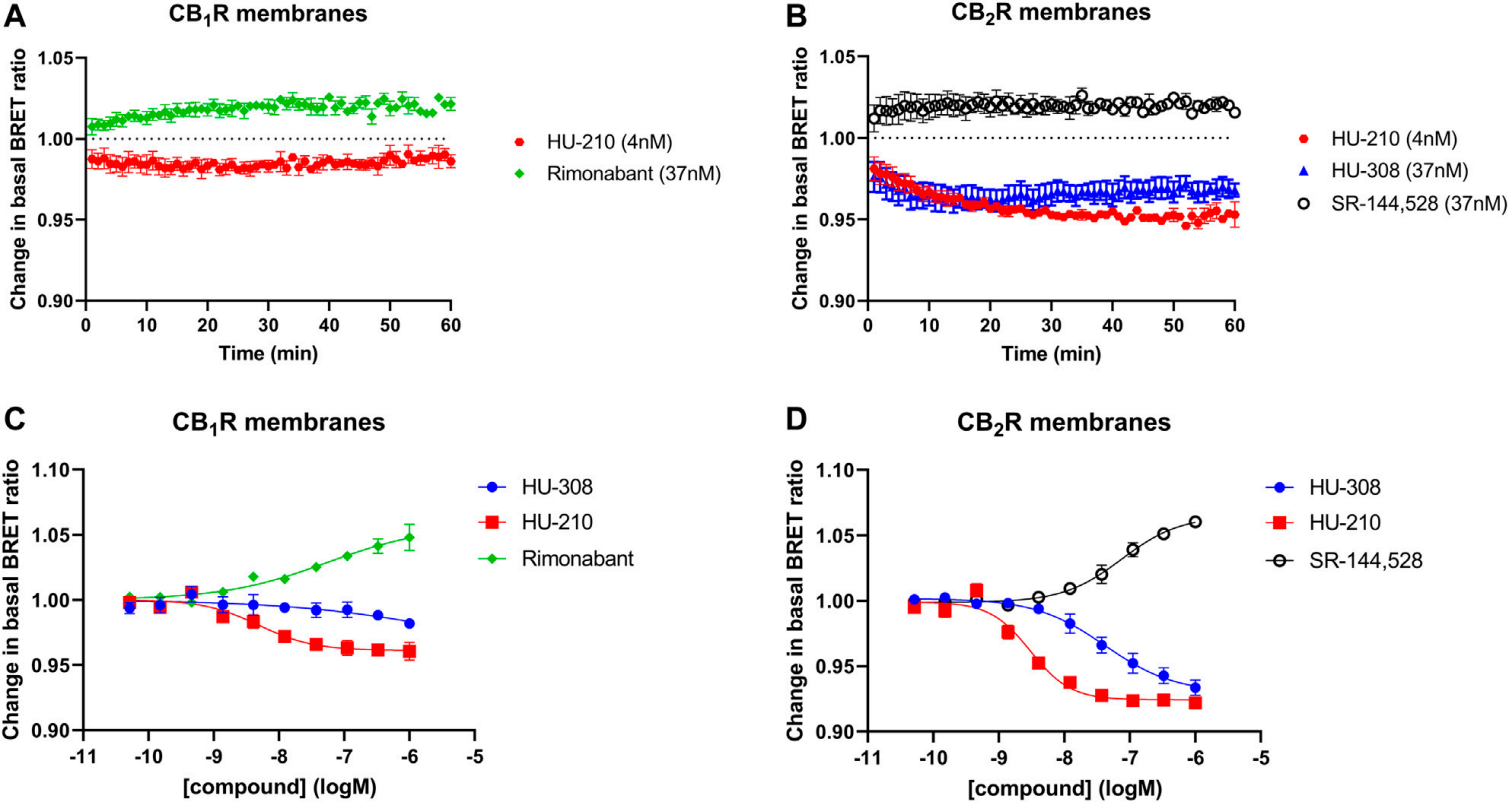
Gi-CASE activation/inhibition time courses and concentration-response curves in CB_1_R- and CB_2_R-expressing HEK293TR membranes, upon stimulation with reference compounds. Time courses at 28°C of **(A)** CB_1_R- or **(B)** CB_2_R-mediated Gi-CASE activation/inhibition following stimulation with HU-210, HU-308 and rimonabant or SR-144,528 at concentrations around the EC_50_. **(C)** CB_1_R- and **(D)** CB_2_R-mediated Gi-CASE concentration-response curves following stimulation with HU-210, HU-308 and rimonabant or SR-144,528. Data are presented as mean ± S.E.M. from three or more experiments.

In general, it is important to maintain a consistent temperature when running functional assays to ensure that the proteins involved remain active. Proteins, particularly enzymes, are sensitive to changes in temperature and can be denatured or lose their activity if the temperature is too high or too low. Running the Gi-CASE assay at a temperature below 30°C helps to ensure that the proteins are active, and the results are reflective of the activation/inactivation process. The Gi-CASE CB_1_R and CB_2_R membrane responses to the four reference ligands were again monitored over time through detection of the BRET signal at 28°C. The response kinetics of the reference agonist and inverse agonist ligands at EC_50_ concentrations at both receptors, measured for up to 60 min at 28°C, are shown in Figures 3A, B. The EC_50_ is a measure of the concentration of a drug, which induces a response halfway between the baseline and maximum. The agonist responses to HU-210 in the CB_1_R membranes appeared more transient than that observed in the CB_2_R membranes. pEC_50_ values of the four reference ligands, measured at 28°C, are summarized in Table 2 and associated response curves are presented in Figures 3C, D.

**TABLE 2:** Membrane-based Gi-CASE signalling assay functional parameters for cannabinoid ligands acting at the human CB_1_ and CB_2_ receptors.

Compound	Membrane based assay							
	Assay temperature, 28°C				Assay temperature, 37°C			
	CB_1_R		CB_2_R		CB_1_R		CB_2_R	
	pEC_50_	E_max_	pEC_50_	E_max_	pEC_50_	E_max_	pEC_50_	E_max_
HU-210	8.48 ± 0.08 (9)	0.954 ± 0.003	8.81 ± 0.09 (10)	0.910 ± 0.005	8.35 ± 0.08 (7)	0.968 ± 0.002	8.55 ± 0.08 (13)	0.908 ± 0.006
HU-308	ND	ND	7.34 ± 0.21 (3)	0.930 ± 0.003	ND	ND	7.97 ± 0.09 (6)	0.926 ± 0.001
Rimonabant	^a^7.16 ± 0.26 (4)	1.056 ± 0.013	ND	ND	^c^7.32 ± 0.26 (3)	1.057 ± 0.003	ND	ND
SR-144,528	ND	ND	^b^7.40 ± 0.27 (3)	1.055 ± 0.008	ND	ND	^b^ ^,^ ^d^7.77 ± 0.31 (6)	1.069 ± 0.011
AM1241	6.08 ± 0.12 (3)	0.975 ± 0.002	7.67 ± 0.02 (3)	0.950 ± 0.002	6.32 ± 0.02 (3)	0.980 ± 0.004	7.69 ± 0.03 (3)	0.944 ± 0.004
JWH-133	5.89 ± 0.16 (3)	0.970 ± 0.02	7.54 ± 0.02 (3)	0.891 ± 0.004	6.31 ± 0.08 (3)	0.982 ± 0.001	7.48 ± 0.03 (3)	0.895 ± 0.004
Cannabinol	6.47 ± 0.10 (3)	0.979 ± 0.002	6.88 ± 0.03 (3)	0.956 ± 0.002	6.51 ± 0.19 (3)	0.980 ± 0.005	6.74 ± 0.06 (3)	0.951 ± 0.004
JTE-907	ND	ND	6.96 ± 0.03 (3)	1.036 ± 0.005	ND	ND	6.99 ± 0.06 (3)	1.028 ± 0.003
CP55,940	8.45 ± 0.07 (3)	0.948 ± 0.002	8.91 ± 0.04 (3)	0.890 ± 0.002	8.28 ± 0.09 (3)	0.972 ± 0.003	8.86 ± 0.02 (3)	0.888 ± 0.000
WIN55212-2	6.75 ± 0.08 (3)	0.950 ± 0.002	8.52 ± 0.03 (3)	0.911 ± 0.002	6.72 ± 0.24 (3)	0.968 ± 0.004	8.47 ± 0.04 (3)	0.901 ± 0.001
AM4113	NE	–	NE	–	NE	–	NE	–

^a^ Rimonabant single shift 7.48 **±** 0.13 (*n* = 4);

^b^ SR-144, 528 single shift 7.78 **±** 0.06 (*n* = 4);

^c^ Rimonabant single shift 7.33 ± 0.10 (*n* = 3);

^d^ SR-144, 528 single shift 7.74 ± 0.05 (*n* = 3).

ND, Not determined; NE, No effect up to 1 μM. Summary table of results from Gi-CASE assays conducted in cannabinoid receptor 1 and 2-expressing HEK293TR cell membranes, in the presence of reference ligands. E_max_ is expressed as change in the basal BRET ratio. Data are averaged (mean ± S.E.M), with number of replicates indicated in parentheses.

**FIGURE 4:**
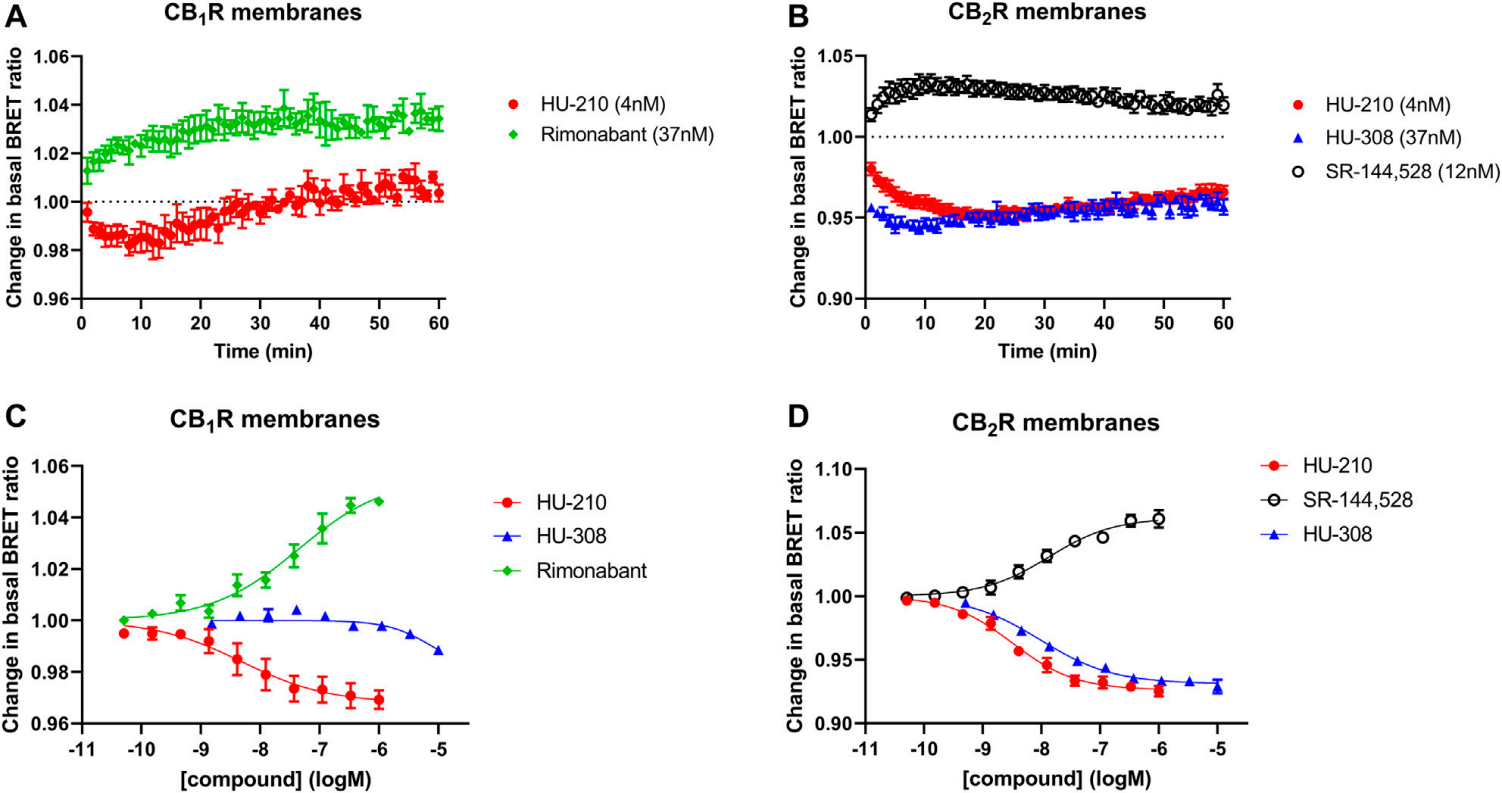
Gi-CASE activation/inhibition time courses and concentration-response curves in CB_1_R- and CB_2_R-expressing HEK293TR membranes, upon stimulation with reference compounds. Time courses at 37°C of **(A)** CB_1_R- or **(B)** CB_2_R-mediated Gi-CASE activation/inhibition following stimulation with HU-210, HU-308 and rimonabant or SR-144,528 at concentrations around the EC_50_. **(C)** CB_1_R- and **(D)** CB_2_R-mediated Gi-CASE concentration-response curves following stimulation with HU-210, HU-308 and rimonabant or SR-144,528. Data are presented as mean ± S.E.M. from three or more experiments.

To directly compare the intact cell and membrane-based responses to the reference ligands under study, we monitored their CB_1_R and CB_2_R Gi-CASE BRET signals at different ligand concentrations at 37°C, as opposed to the 28°C measurements described above. The response kinetics of reference agonist and inverse agonist ligands at EC_50_ concentrations were measured for up to 60 min at 37°C, are shown in Figures 4A, B. Both CB_1_R and CB_2_R membrane-derived BRET responses were more transient compared to those measured at 28°C. pEC_50_ values of the four reference ligands measured at 37°C, are summarized in Table 2 and associated response curves are presented in Figures 4C, D.

**FIGURE 5:**
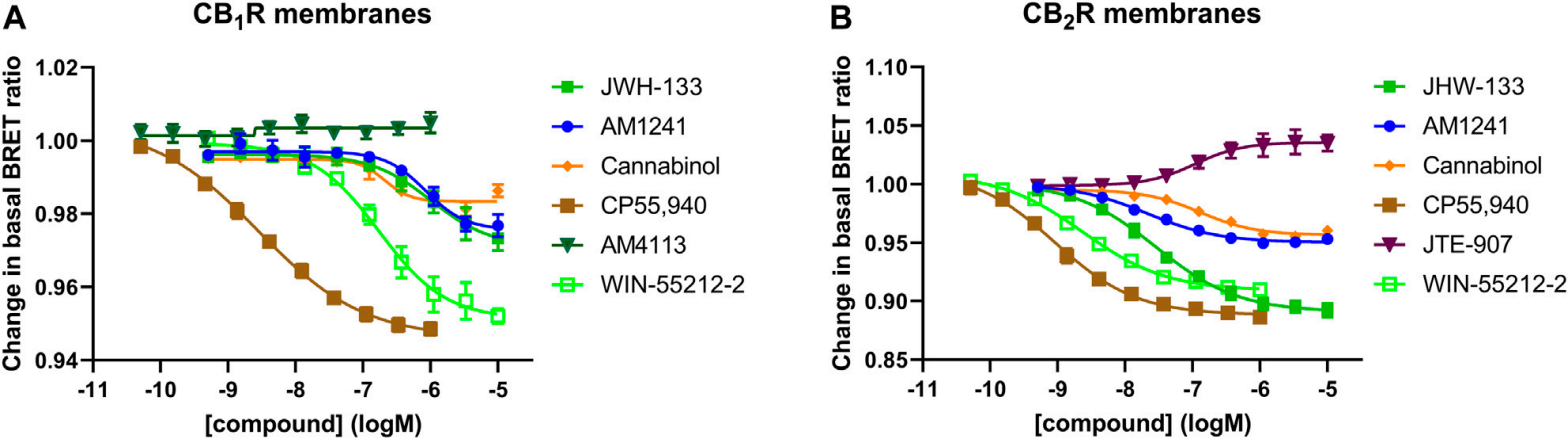
Gi-CASE activation/inhibition concentration-response curves in CB_1_R- and CB_2_R-expressing HEK293TR membranes, upon stimulation with cannabinoid agonists, inverse agonists and neutral compounds. Gi-CASE mediated concentration-response curves obtained in membranes expressing **(A)** CB_1_R and **(B)** CB_2_R. Data are presented as mean ± S.E.M. from three or more experiments.

**FIGURE 6:**
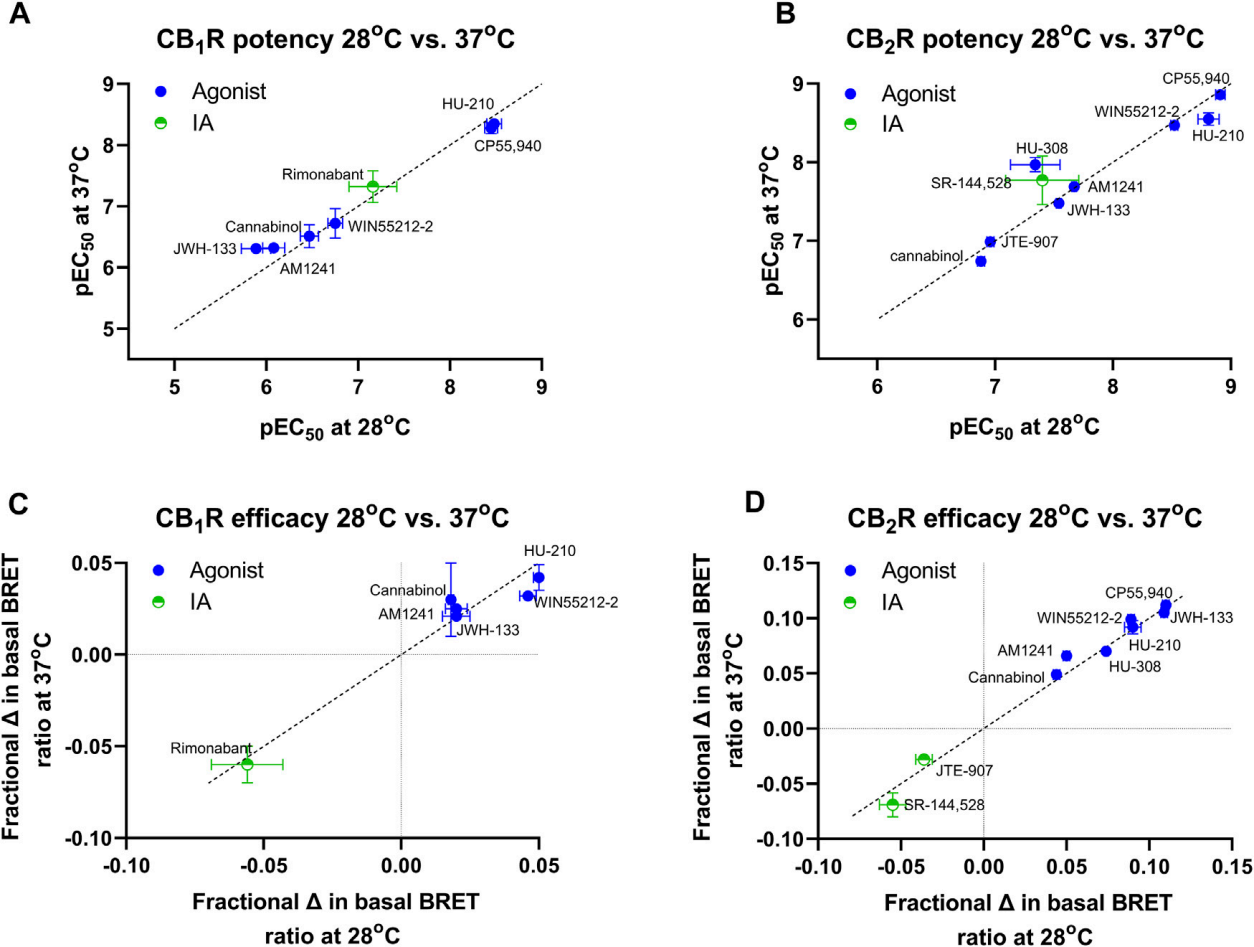
A comparison of CB_1_R and CB_2_R activation/inhibition potency and efficacy estimates for test compounds obtained at 28°C and 37°C in the Gi-CASE membrane assay. Compound pEC_50_ value comparison in **(A)** CB_1_R- and **(B)** CB_2_R-expressing Gi-CASE membranes at 28°C and 37°C. Compound intrinsic activity (efficacy) measure comparison expressed as fractional change in the basal BRET ratio in **(C)** CB_1_R- and **(D)** CB_2_R-expressing Gi-CASE membranes at 28°C and 37°C. The dashed line indicates the unity line for perfect correlation. Data are presented as mean ± S.E.M. from three or more experiments.

**FIGURE 7:**
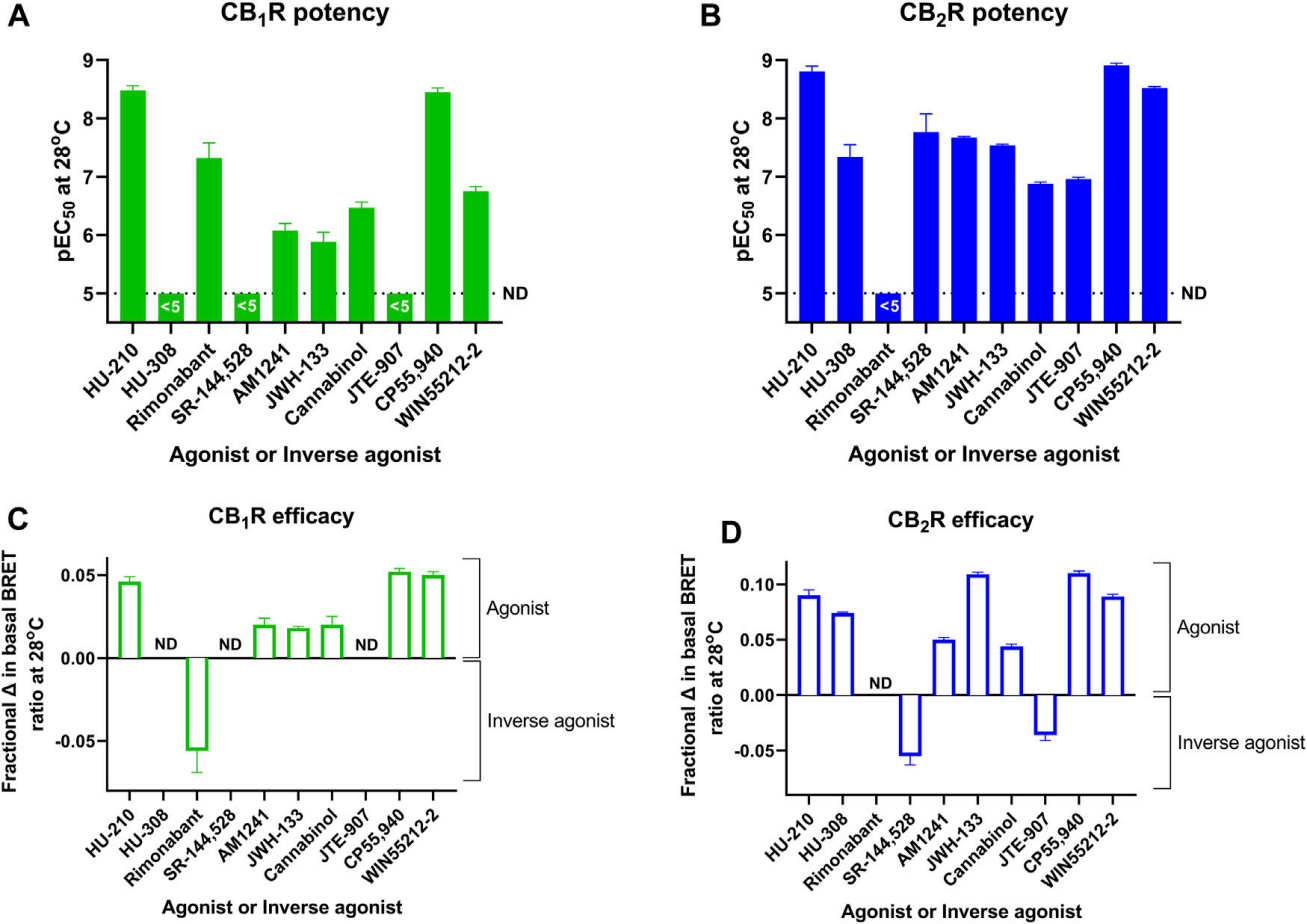
A direct comparison of CB_1_R and CB_2_R activation/inhibition potency and efficacy estimates for test compounds obtained at 28°C in the Gi-CASE membrane assay. Compound pEC_50_ values obtained in **(A)** CB_1_R- and **(B)** CB_2_R-expressing Gi-CASE membranes. Compound intrinsic activity measures (efficacy) expressed as fractional change in the basal BRET ratio at **(C)** CB_1_R and **(D)** CB_2_R. The dashed line in A and B is the lower limit of potency detection with values below this level not determined (ND). Data are presented as mean ± S.E.M. from three or more experiments.

In a series of further tests, we profiled a collection of ligands known to bind and activate CB_1_R and CB_2_R with varying degrees of potency and efficacy. The results of these tests performed at 28°C and 37°C are shown in Figures 5A, B. A comparison of ligand potency and efficacy determined at 28°C and 37°C at both CB_1_R and CB_2_R is shown in Figures 6A–D. These figures highlight the similarities in compound potency and efficacy estimates obtained at 28°C and 37°C. In view of the more stable inactivation/activation responses obtained in time course experiments obtained at 28°C, we would recommend future profiling of potential CB_1_R and CB_2_R compounds at this lower temperature. A comparison of individual compound potency and efficacy measurements at CB_1_R and CB_2_R obtained at 28°C is shown in Figures 7A–D, respectively. Compound pEC_50_ and maximal response values (expressed as fractional change in basal response) are detailed in Table 2.

**FIGURE 8:**
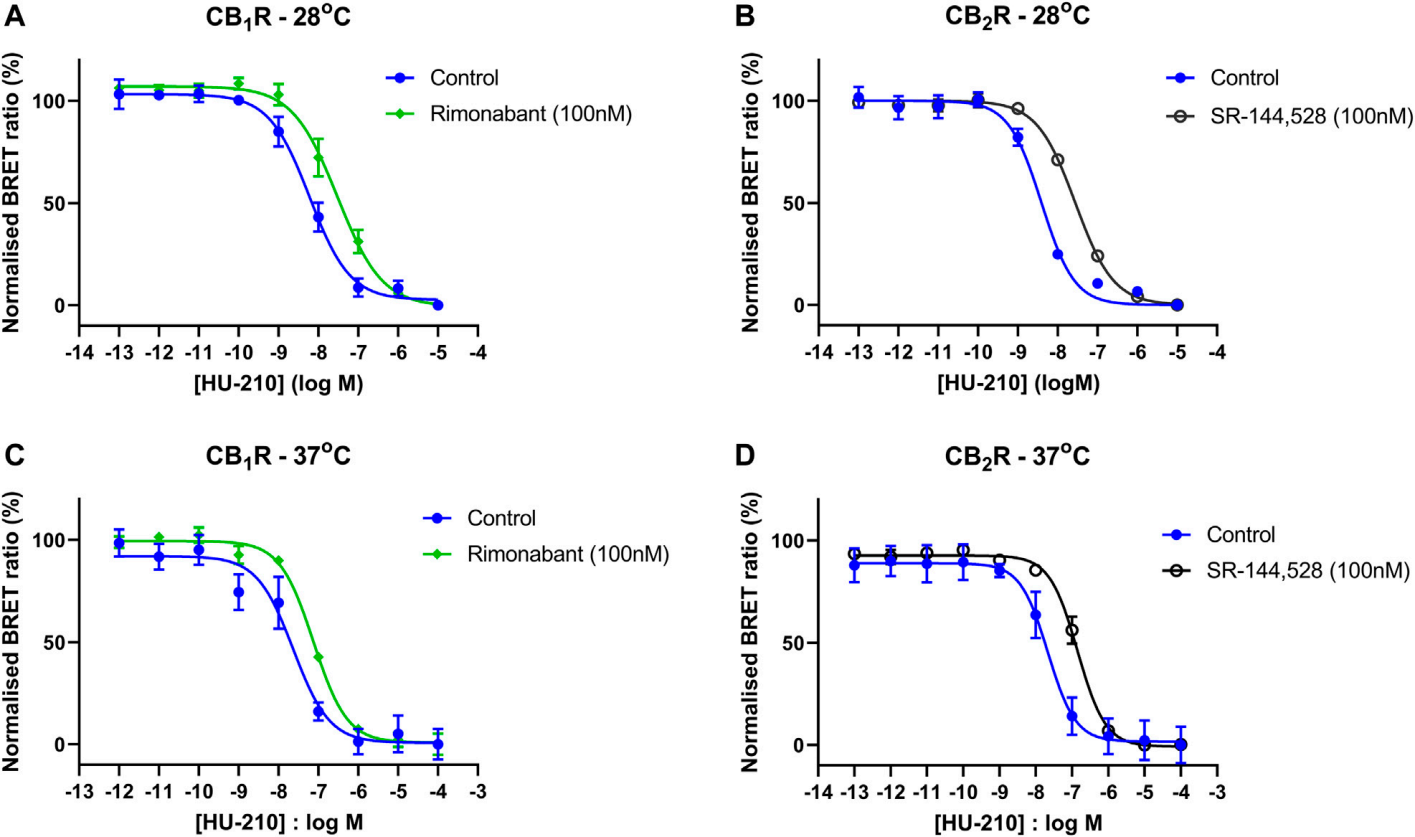
Inverse agonist-induced shifts in CB_1_R- and CB_2_R-expressing HEK293TR membranes. HU-210 Gi-CASE concentration-response curves obtained in HEK293TR **(A)** CB_1_R membranes at 28°C, in the absence and presence of the reference CB_1_R inverse agonist rimonabant and **(B)** CB_2_R membranes at 28°C, in the absence and presence of the reference CB_1_R inverse agonist SR-144,528 **(C)** CB_1_R membranes at 37°C, in the absence and presence of rimonabant and **(D)** CB_2_R membranes at 37°C, in the absence and presence of SR-144,528. Gi-CASE response data are presented as mean ± S.E.M. from three separate experiments performed in singlet.

To better characterize the selective inverse agonists rimonabant and SR-144,528, we conducted single-shift experiments at 28°C and 37°C to derive affinity values. The two test ligands were preincubated with CB_1_R and CB_2_R membranes prior to their addition to assay wells containing the reference agonist HU-210. The results of these single shift experiments in CB_1_R and CB_2_R membranes are shown in Figure 8 and the resulting pA_2_ estimates for each ligand are stated in the text under Table 2.

### Membrane-based Gi-CASE CB_2_R cumulative addition experiments

**FIGURE 9:**
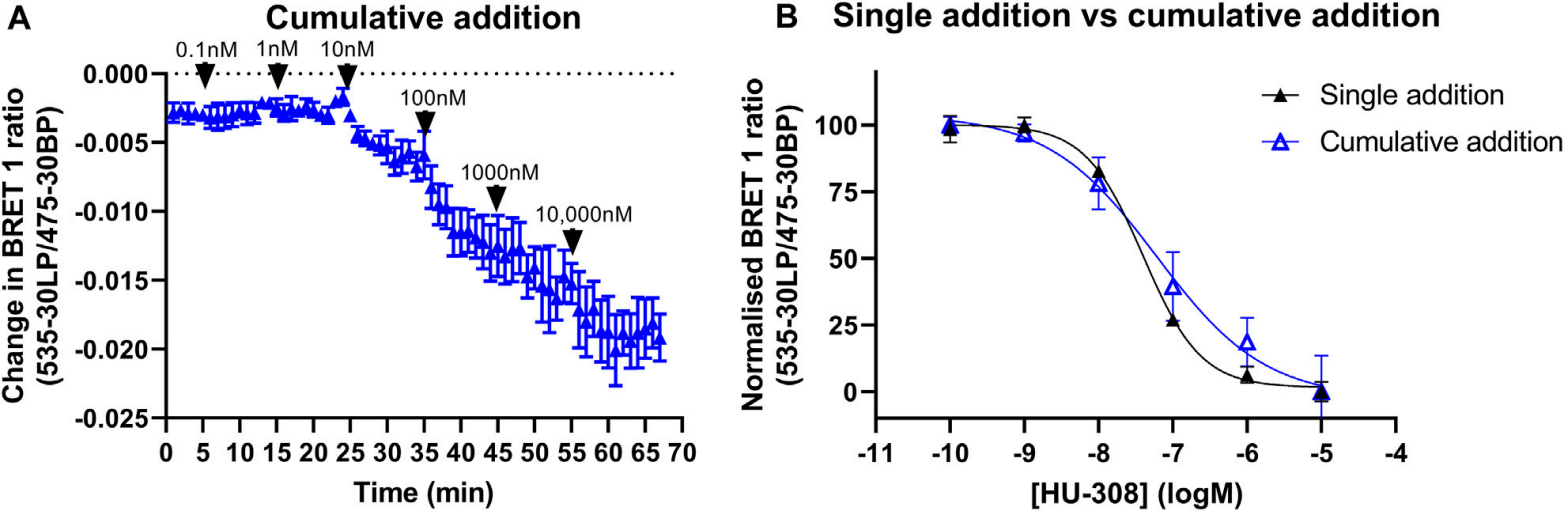
Gi-CASE activation time courses and concentration-response curves in CB_2_R-expressing HEK293TR membranes, upon stimulation with the reference agonist HU-308. Changes in Gi-CASE biosensor activation was detected by BRET in HEK293TR membranes. **(A)** Consecutive addition of increasing concentrations of HU-308 into the same assay wells. Data are displayed as mean ± S.E.M. from three experiments performed in triplicate. **(B)** Concentration-response curve of HU-308 generated from **(A)**; 10 min after each consecutive injection of increasing HU-308 concentrations (triplicate determinations), or 60 min after stimulation of individual wells with increasing concentrations HU-308 (singlet determinations). Data are displayed as mean ± S.E.M. from three independent experiments.

Increasing concentrations of HU-308 added to the same well with 10-minute time intervals resulted in a concentration-dependent decrease in the BRET signal (Figure 9A). The concentration-response curve (pEC_50_ = 7.23 ± 0.17) generated from the change in BRET ratios that were taken 10 min after each consecutive injection and had a comparable amplitude to the concentration-response curve obtained from individual wells, that were each stimulated with a different HU-308 concentration (pEC_50_ = 7.39 ± 0.03, Figure 9B).

### Relationship between membrane-based and intact cell-based Gi-CASE and literature compound potency values

**FIGURE 10:**
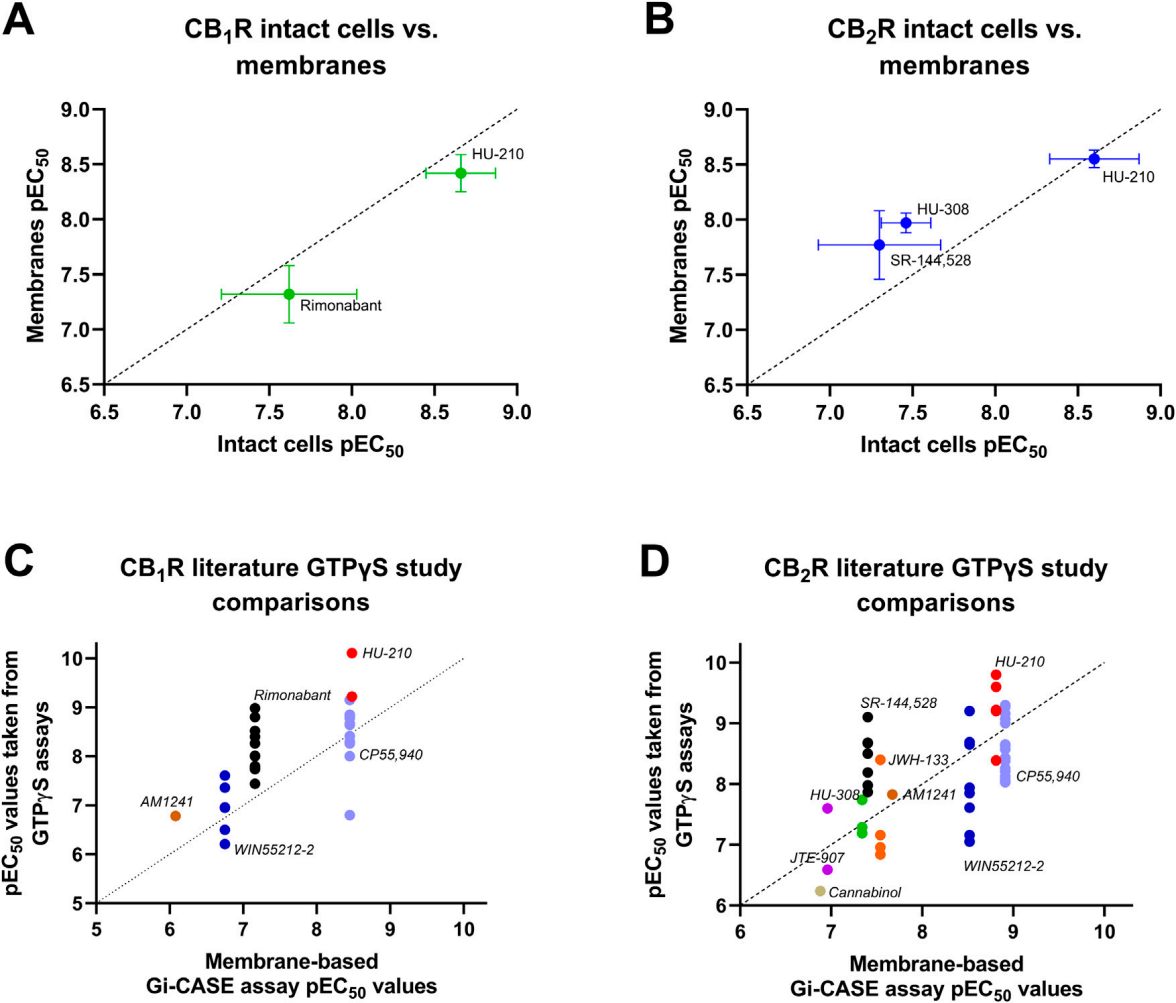
Relationships between Gi-CASE and literature compound pEC_50_ values. Correlation plots of Gi-CASE assay in intact cell and membrane-based assay compound pEC_50_ values at **(A)** CB_1_R and **(B)** CB_2_R. Comparison of membrane-assay format derived **(C)** CB_1_R and **(D)** CB_2_R Gi-CASE compound pEC_50_ values, obtained at 28°C, and literature derived pEC_50_ values obtained in the commonly used GTPγS binding assay format (the source of the GTPγS binding data is provided in the Supplementary File). All Gi-CASE pEC_50_ values were taken from experiments shown in Figures 2, 4, 5. All data used in these plots are detailed in Table 1, 2. Data are presented as mean ± S.E.M. from three or more experiments. The dashed line indicates the unity line for perfect correlation.

A comparison was made between whole-cells and membranes expressing the Gi-CASE biosensor and either CB_1_R or CB_2_R, to illustrate the relationship between the two assay formats (see Figures 10A, B). There was a very good correlation between pEC_50_ values obtained at both receptors, indicating that the membrane-derived parameters are largely comparable with the intact cell parameters measured at 37°C. A further comparison was made between the membrane-based assay format-derived CB_1_R and CB_2_R Gi-CASE pEC_50_ values obtained at 28°C and literature derived pEC_50_ values, obtained in the commonly used GTPγS binding assay format. Correlations of assay-derived compound pEC_50_ values at the two receptor subtypes are shown in Figures 10C, D. In general, there was strong positive correlation between the compound pEC_50_ values obtained in CB_1_R membrane-based Gi-CASE assay format and literature-derived GTPγS binding pEC_50_ values. For CB_2_R there was a moderate positive correlation derived from the literature-derived GTPγS binding assay and the membrane-based Gi-CASE assay pEC_50_ values (Pearson’s correlation for CB_1_R, *p* = 0.03 and the correlation coefficient, *r* = 0.91, and for CB_2_R, *p* = 0.01 and *r* = 0.80). In terms of observed compound efficacy at CB_2_R, the higher efficacy ligands can be ranked as follows, moving from high to low efficacy: CP55,940>JWH-133>HU-210>WIN55212-2>HU-308 (see Figure 7D). These observed differences in compound efficacy at the CB_2_R of these more efficacious compounds is broadly similar to other GTPγS binding studies where one or more of these ligands has been compared directly (Govaerts et al., 2004; Stern et al., 2006; Smoum et al., 2015; Soethoudt et al., 2017). A single study looking at CB_1_R compound efficacy also shows CP55,940, HU-210 and WIN55212-2 behaving as full agonists with practically indistinguishable levels of intrinsic activity (Govaerts et al., 2004).

## Discussion

Historically, Gα_i_-based signalling is often investigated at a single time point by monitoring cAMP inhibition in intact cellular systems, or directly by using membranes via [^35^S]-GTPγS binding. To address the limitations of the existing assays that monitor Gα_i_ activation, we have developed a simple membrane-based assay that uses BRET-based biosensors to measure Gα_i_ protein dissociation upon activation by the receptor. By using a single Gi-CASE plasmid for the introduction of fluorescently active Gα_i_βγ protein, we reduce the variation in individual G protein subunit expression levels, which positively impacts assay sensitivity. One of the significant benefits of using novel biosensors is that they enable us to study the kinetics of G protein activation in real-time (Olsen and English, 2022).

All compounds profiled in the Gi-CASE assays showed expected behaviour. For example, activation of CB_1_R and CB_2_R in both our whole-cell and membrane-based Gi-CASE NanoBRET systems by the non-selective reference agonist HU-210, resulted in a decrease in the BRET ratio, with observed pEC_50_ values in the low nM range. This is consistent with the literature-derived GTPγS binding assay values obtained under similar conditions (Muccioli et al., 2006; Manera et al., 2015). Similarly, HU-308, a CB_2_R specific agonist, activated the CB_2_R with nM potency, with some effects observed at the CB_1_R but only in the high μM range. These findings are very much in line with its greater selectivity for the CB_2_R over CB_1_R, and previous functional studies (Soethoudt et al., 2017).

Rimonabant is a CB_1_R-specific blocker, which binds with a much higher affinity to the human CB_1_R compared to CB_2_R (Pettersson et al., 2009). In the current study, rimonabant acted as a strong inverse agonist of CB_1_R function in both intact cell and membrane assay formats, inhibiting Gα_i_ dissociation from Gβγ, resulting in an increase in the BRET ratio and a pEC_50_ value of 7.16 at 28°C. Similar potency values for rimonabant acting on CB_1_R were reported in the original paper by Schihada and colleagues, which used intact cells (Schihada et al., 2021). Shift experiments, conducted at 28°C and 37°C, predict a pA_2_ of rimonabant in line with those obtained in the direct activation assays where inverse agonism was observed (see Table 2).

SR-144,528 is a classically used CB_2_R-specific inverse agonist, which binds with a much higher affinity for hCB_2_R, than CB_1_R (Rinaldi-Carmona et al., 1998; Portier et al., 1999). Acting at the CB_2_R in the current system, SR-144,528, akin to rimonabant acting at CB_1_R, promotes the accumulation of the Gα_i_βγ heterotrimer, resulting in an increase in the BRET ratio. The functional potency of SR-144,528 for the CB_2_R, determined using [^35^S]-GTPγS binding, cAMP and β-arrestin assay formats, has revealed pEC_50_ values of 7.87, 7.67 and 7.47, respectively, which are similar to the pEC_50_ value of 7.77 and the single shift pA_2_ value of 7.78, observed in the current study at 28°C (Soethoudt et al., 2017).

The level of functional activation of a GPCR can be influenced by variations in assay conditions, including receptor density and the availability of signal transduction molecules such as G proteins. In our current system, we can control receptor density through tetracycline induction and can vary G protein levels by selecting clones with different expression levels of Venus-labelled G protein. In the absence of an agonist, the conformation of a GPCR spontaneously transitions between the inactive (R) and active (R*) states, in a process referred to as basal or constitutive activity. This so-called ligand-independent receptor activation results in an observable increase in the dissociation of the heterotrimeric G protein complex in the absence of any ligand. When an agonist binds to the orthosteric site on a GPCR, it will promote the formation of the active receptor state, R*. In contrast, an inverse agonist will reduce basal activity by shifting the equilibrium towards the inactive state, R. Neutral antagonists can also exist, which bind to the orthosteric site, but leave the equilibrium between active and inactive states unaltered. Our membrane preparations for CB_1_R and CB_2_R are consistent with this 2-state model and a shift in the balance of cannabinoid receptors from the coupled on-state to the coupled off-state in the presence of the inverse agonists, rimonabant and SR-144,528.

In our current system, AM1241 behaved as a partial agonist at both CB_1_R and CB_2_R. In previous reports, AM1241 has been shown to behave as a protean agonist at hCB_2_R, activity which is only revealed after abolition of constitutive receptor activity (Mancini et al., 2009). Similar findings have been observed with other protean ligands when cholesterol levels are altered (Yeliseev et al., 2021). Protean agonists can exhibit different pharmacology and downstream effects depending on the system they are studied in and the specific receptor and signalling pathways involved, as well as the conformational changes induced by the ligand-receptor interaction. The term ‘protean’ reflects the fact that these agonists can manifest a wide range of effects, often making their pharmacology complex and difficult to predict.

In theory, any factor increasing the constitutive activity of a receptor should produce an increase in inverse agonist efficacy and a decrease in agonist efficacy (Bosier et al., 2010; Marini et al., 2013). As such, small changes in assay conditions can play a significant role in dictating ligand affinity and potency measurements. For example, sodium ions and cholesterol are known to regulate the active conformations of certain GPCRs, including cannabinoid receptors (Showalter et al., 1996; Chini and Parenti, 2009; Agasid et al., 2021). These modulatory factors will in turn influence the binding of the GPCR to G proteins through alterations in the seven transmembrane helices, and as a direct consequence of this, any observable agonist responses (Liu et al., 2012; Manglik and Kruse, 2017; Yeliseev et al., 2021). Precise control over these assay variables may allow for the discovery of drug-specific active states, a feature of protean agonists, which may be beneficial in certain clinical situations (Kenakin, 2001).

AM4113 is a reported CB_1_R neutral antagonist which exhibits nM potency at the hCB_1_R (Sink et al., 2008). AM4113 did not alter basal Gi-CASE activation in the membrane-based system, confirming the suitability of the Gi-CASE assay, in combination with binding assays, for separating out ligands of diverging efficacy to target the CB_1_R. This finding also implies that endogenous agonists are not present in the current CB_1_R system. The discovery of neutral CB_1_R antagonists for the treatment of substance use disorders has gained some interest in recent years, highlighting the utility of this new assay format, which can seemingly distinguish between molecules with divergent signalling efficacy (AlKhelb et al., 2022; Soler-Cedeno and Xi, 2022). Neutral receptor antagonists are expected to produce inverse effects through antagonism of endogenously released endocannabinoids but not by modulating CB_1_R constitutive activity. Part of the promise of neutral antagonists stems from their reduced effects on basal CBR signalling, which should in theory result in overall reduced systemic inflammation (O'Keefe et al., 2022) and a reduction in rimonabant-like side effects which can include nausea, malaise and anxiety (Chambers et al., 2007; Jarbe et al., 2008; Sink et al., 2008; Gueye et al., 2016).

The full potential of the membrane-based Gi-CASE functional assay for screening large chemical compound libraries against the CB_1_R and CB_2_R subtypes is highlighted in Figures 5–7. With consideration to biased signalling, the current membrane-based Gi-CASE system allows for the robust quantification of weak agonist and inverse agonist effects, both in terms of potency and intrinsic activity. Importantly, Gi-CASE activity data is wholly consistent with previous reports which have demonstrated CP55,940 to be a non-selective full agonist at both CB_1_R and CB_2_R (Dziadulewicz et al., 2007; Diaz et al., 2009; Ouyang et al., 2013), and WIN55212-2 and JWH-133 to be high efficacy CB_2_R selective agonists (Fichera et al., 2000; Manera et al., 2006; Salo et al., 2006; Stern et al., 2006; Dziadulewicz et al., 2007; Navarro et al., 2018). Cannabinol appears to be a nonselective, weak CB_1_R and CB_2_R partial agonist in line with previous findings at the CB_1_R and CB_2_R (Showalter et al., 1996; Rhee et al., 1997), whilst AM1241, which is also a partial agonist, appears to be more selective for CB_2_R (Harnett et al., 2015). JTE-907 appears to be a CB_2_R selective inverse agonist but with a reduced efficacy compared to SR-144,528, a finding consistent with previous reports on this ligand (Iwamura et al., 2001).

The Gi-CASE compound time course data, along with potency and intrinsic activity measures collected in this study, suggests that 28°C is a suitable temperature to perform future cannabinoid screens. The temperature insensitivity of compound responsiveness, as assessed by measures of potency and maximal response, suggests that receptor-effector coupling is not significantly affected by factors such as lateral diffusion or encounter rate across this range of temperatures. This implies that all essential components for efficient and measurable receptor-G protein coupling are present and functioning optimally. The close agreement between the pEC_50_ values obtained from our membrane-based Gi-CASE assay and those derived from intact cells indicates that the former system provides biologically relevant estimates of compound potency and activity (see Figures 10A, B).

Our study demonstrates that the 384-well NanoBRET Gi-CASE membrane-based assay is a reliable and valuable tool for characterizing both CB_1_R and CB_2_R agonist and inverse agonist activity. Compared to intact cell-based assays, membrane-based assays offer reduced test-to-test variation and several advantages for high-throughput screening, such as the ability to prepare a single homogenous batch of membranes for on-demand use. Additionally, the use of membranes eliminates the need for wash and furimazine incubation steps, which are potential sources of assay variation in other formats of the Gi-CASE assay.

## Conclusion

In summary, we have developed a novel membrane-based functional assay that utilizes existing Gi-CASE biosensors designed for monitoring GPCR activation in intact cellular systems, such as those involving CBRs. Our membrane-based NanoBRET assay is more efficient and amenable to automation compared to current assay formats. It enables high-throughput screening of novel agonists and inverse agonists targeting CB_1_R and CB_2_R, as well as the identification of ligands for orphan GPCRs that couple to Gα_i_ proteins. Overall, this innovative assay represents a promising tool for advancing drug discovery efforts targeting GPCR-mediated signalling pathways.

## Data Availability

The raw data supporting the conclusion of this article will be made available by the authors, without undue reservation.

## Abbreviations

HEK293-TREX, Human embryonic kidney tetracycline repressor; HBSS, Hanks Balanced Salt Solution; Gi-CASE, Gα_i_ protein tricistronic activity sensor; EC_50_, concentration of half maximal effective concentration; IA, Inverse agonist.
