# Supplementary material for: Development of a membrane-based Gi-CASE biosensor assay for profiling compounds at cannabinoid receptors
Source: Front Pharmacol. 2023 Aug 11;14:1158091. doi: 10.3389/fphar.2023.1158091 (PMC10450933; doi:10.3389/fphar.2023.1158091)
Supplement: Supplementary file 1 [file Table1.DOCX]

**Supplementary information**

Literature GTPγS binding assay pEC_50_ values for the ligands under study in the Gi-CASE assay.

| **Compound** | **hCB1R pEC50** | **Reference** | **hCB2R pEC50** | **Reference** |
| --- | --- | --- | --- | --- |
| **SR144,528** |  |  | 7.98 | https://pubmed.ncbi.nlm.nih.gov/10188977/ |
|  |  |  | 8.19 | https://pubmed.ncbi.nlm.nih.gov/23711022/ |
|  |  |  | 8.68 | https://pubmed.ncbi.nlm.nih.gov/23151320/ |
|  |  |  | 9.10 | https://pubmed.ncbi.nlm.nih.gov/17245363/ |
|  |  |  | 8.50 | https://pubmed.ncbi.nlm.nih.gov/17177896/ |
|  |  |  | 8.68 | https://pubmed.ncbi.nlm.nih.gov/16392793/ |
|  |  |  | 8.67 | https://pubmed.ncbi.nlm.nih.gov/15489124/ |
|  |  |  | 7.87 | https://pubmed.ncbi.nlm.nih.gov/28045021/ |
|  |  |  |  |  |
|  |  |  | **MEAN** | **SEM** |
|  |  |  | **8.46** | **0.15** |

| **Compound** | **hCB1R pEC50** | **Reference** | **hCB2R pEC50** | **Reference** |
| --- | --- | --- | --- | --- |
| **Rimonabant** | 8.40 | https://pubmed.ncbi.nlm.nih.gov/19520572/ |  |  |
|  | 8.00 | https://pubmed.ncbi.nlm.nih.gov/16451053/ |  |  |
|  | 7.74 | https://pubmed.ncbi.nlm.nih.gov/18712856/ |  |  |
|  | 8.80 | https://pubmed.ncbi.nlm.nih.gov/19351113/ |  |  |
|  | 7.78 | https://pubmed.ncbi.nlm.nih.gov/18219411/ |  |  |
|  | 7.44 | https://pubmed.ncbi.nlm.nih.gov/29793210/ |  |  |
|  | 8.01 | https://pubmed.ncbi.nlm.nih.gov/29229226/ |  |  |
|  | 8.52 | https://pubmed.ncbi.nlm.nih.gov/17433696/ |  |  |
|  | 7.74 | https://pubmed.ncbi.nlm.nih.gov/18712856/ |  |  |
|  | 8.00 | https://pubmed.ncbi.nlm.nih.gov/16279809/ |  |  |
|  | 7.80 | https://pubmed.ncbi.nlm.nih.gov/19530697/ |  |  |
|  | 8.26 | https://pubmed.ncbi.nlm.nih.gov/9690851/ |  |  |
|  | 8.98 | https://pubmed.ncbi.nlm.nih.gov/28045021/ |  |  |
|  |  |  |  |  |
|  | **MEAN** | **SEM** |  |  |
|  | **8.11** | **0.13** |  |  |

| **Compound** | **hCB1R pEC50** | **Reference** | **hCB2R pEC50** | **Reference** |
| --- | --- | --- | --- | --- |
| **HU308** |  |  | 7.74 | https://www.ebi.ac.uk/chembl/document_report_card  /CHEMBL3886783/ |
|  |  |  | 7.29 | https://pubmed.ncbi.nlm.nih.gov/28045021/ |
|  |  |  | 7.19 | https://pubmed.ncbi.nlm.nih.gov/27309150/ |
|  |  |  |  |  |
|  |  |  | **MEAN** | **SEM** |
|  |  |  | **7.41** | **0.17** |

| **Compound** | **hCB1R pEC50** | **Reference** | **hCB2R pEC50** | **Reference** |
| --- | --- | --- | --- | --- |
| **HU-210** | 9.22 | https://pubmed.ncbi.nlm.nih.gov/15489124/ | 9.2 | https://pubmed.ncbi.nlm.nih.gov/25935384/ |
|  | 10.11 | https://pubmed.ncbi.nlm.nih.gov/25447744/ | 9.22 | https://pubmed.ncbi.nlm.nih.gov/15489124/ |
|  |  |  | 9.2 | https://pubmed.ncbi.nlm.nih.gov/25447744/ |
|  |  |  | 8.39 | https://pubmed.ncbi.nlm.nih.gov/16392793/ |
|  |  |  | 9.80 | https://pubmed.ncbi.nlm.nih.gov/15852035/ |
|  |  |  | 9.60 | https://pubmed.ncbi.nlm.nih.gov/16279774/ |
|  |  |  |  |  |
|  | **MEAN** | **SEM** | **MEAN** | **SEM** |
|  | **9.67** |  | **9.23** | **0.20** |

| **Compound** | **hCB1R pEC50** | **Reference** | **hCB2R pEC50** | **Reference** |
| --- | --- | --- | --- | --- |
| **CP55,940** | 6.8 | https://pubmed.ncbi.nlm.nih.gov/17027269/ | 8.57 | https://pubmed.ncbi.nlm.nih.gov/18666769/ |
|  | 8.00 | https://pubmed.ncbi.nlm.nih.gov/19115816/ | 8.06 | https://pubmed.ncbi.nlm.nih.gov/19115816/ |
|  | 8.26 | https://pubmed.ncbi.nlm.nih.gov/29793210/ | 8.26 | https://pubmed.ncbi.nlm.nih.gov/29793210/ |
|  | 8.78 | https://pubmed.ncbi.nlm.nih.gov/32340793/ | 8.03 | https://pubmed.ncbi.nlm.nih.gov/25072877/ |
|  | 8.82 | https://pubmed.ncbi.nlm.nih.gov/33915369/ | 8.38 | https://pubmed.ncbi.nlm.nih.gov/24125850/ |
|  | 8.84 | https://pubmed.ncbi.nlm.nih.gov/25065940/ | 9.27 | https://pubmed.ncbi.nlm.nih.gov/33915369/ |
|  | 8.41 | https://pubmed.ncbi.nlm.nih.gov/15489124/ | 8.63 | https://pubmed.ncbi.nlm.nih.gov/25065940/ |
|  | 8.85 | https://pubmed.ncbi.nlm.nih.gov/31756109/ | 9 | https://pubmed.ncbi.nlm.nih.gov/15489124/ |
|  | 9.15 | https://pubmed.ncbi.nlm.nih.gov/17919913/ | 9.07 | https://pubmed.ncbi.nlm.nih.gov/18006322/ |
|  | 9.15 | https://pubmed.ncbi.nlm.nih.gov/23085772/ | 9.16 | https://pubmed.ncbi.nlm.nih.gov/17919913/ |
|  | 8.67 | https://pubmed.ncbi.nlm.nih.gov/9690851/ | 8.07 | https://pubmed.ncbi.nlm.nih.gov/23711022/ |
|  |  |  | 8.13 | https://pubmed.ncbi.nlm.nih.gov/23085772/ |
|  |  |  | 8.65 | https://pubmed.ncbi.nlm.nih.gov/9690851/ |
|  |  |  | 8.21 | https://pubmed.ncbi.nlm.nih.gov/16392793/ |
|  |  |  | 9.3 | https://pubmed.ncbi.nlm.nih.gov/15852035/ |
|  |  |  |  |  |
|  | **MEAN** | **SEM** | **MEAN** | **SEM** |
|  | **8.51** | **0.17** | **8.59** | **0.12** |

| **Compound** | **hCB1R pEC50** | **Reference** | **hCB2R pEC50** | **Reference** |
| --- | --- | --- | --- | --- |
| **JWH-133** | 7.37 | https://pubmed.ncbi.nlm.nih.gov/28045021/ | 8.40 | https://www.ebi.ac.uk/chembl/document_report_card  /CHEMBL3886783/ |
|  |  |  | 6.84 | https://pubmed.ncbi.nlm.nih.gov/28045021/ |
|  |  |  | 7.16 | https://pubmed.ncbi.nlm.nih.gov/27309150/ |
|  |  |  | 6.96 | https://pubmed.ncbi.nlm.nih.gov/28045021/ |
|  |  |  |  |  |
|  | **MEAN** |  | **MEAN** | **SEM** |
|  | **7.37** |  | **7.34** | **0.46** |

| **Compound** | **hCB1R pEC50** | **Reference** | **hCB2R pEC50** | **Reference** |
| --- | --- | --- | --- | --- |
| **WIN55212-2** | 6.5 | https://pubmed.ncbi.nlm.nih.gov/17027269/ | 7.05 | https://pubmed.ncbi.nlm.nih.gov/23017078/ |
|  | 6.95 | https://pubmed.ncbi.nlm.nih.gov/22607668/ | 7.61 | https://pubmed.ncbi.nlm.nih.gov/20979417/ |
|  | 6.96 | https://pubmed.ncbi.nlm.nih.gov/23227781/ | 7.85 | https://pubmed.ncbi.nlm.nih.gov/18522867/ |
|  | 7.36 | https://pubmed.ncbi.nlm.nih.gov/27309150/ | 7.94 | https://pubmed.ncbi.nlm.nih.gov/30583970/ |
|  | 6.21 | https://pubmed.ncbi.nlm.nih.gov/9690851/ | 8.69 | https://pubmed.ncbi.nlm.nih.gov/23849204/ |
|  | 7.61 | https://pubmed.ncbi.nlm.nih.gov/28045021/ | 7.61 | https://pubmed.ncbi.nlm.nih.gov/16392793/ |
|  |  |  | 7.16 | https://pubmed.ncbi.nlm.nih.gov/23711022/ |
|  |  |  | 7.05 | https://pubmed.ncbi.nlm.nih.gov/23151320/ |
|  |  |  | 8.65 | https://pubmed.ncbi.nlm.nih.gov/9690851/ |
|  |  |  | 9.2 | https://pubmed.ncbi.nlm.nih.gov/15852035/ |
|  |  |  | 7.96 | https://pubmed.ncbi.nlm.nih.gov/28045021/ |
|  |  |  |  |  |
|  | **MEAN** | **SEM** | **MEAN** | **SEM** |
|  | **6.80** | **0.20** | **7.89** | **0.22** |

| **Compound** | **hCB1R pEC50** | **Reference** | **hCB2R pEC50** | **Reference** |
| --- | --- | --- | --- | --- |
| **Cannabinol** |  |  | 6.24 | https://pubmed.ncbi.nlm.nih.gov/9690851/ |
|  |  |  |  |  |
|  |  |  | **MEAN** | **SEM** |
|  |  |  | **6.24** |  |

| **Compound** | **hCB1R pEC50** | **Reference** | **hCB2R pEC50** | **Reference** |
| --- | --- | --- | --- | --- |
| **JTE-907** |  |  | 7.60 | https://pubmed.ncbi.nlm.nih.gov/17177896/ |
|  |  |  | 6.59 | https://pubmed.ncbi.nlm.nih.gov/23711022/ |
|  |  |  |  |  |
|  |  |  | **MEAN** | **SEM** |
|  |  |  | **7.10** |  |

| **Compound** | **hCB1R pEC50** | **Reference** | **hCB2R pEC50** | **Reference** |
| --- | --- | --- | --- | --- |
| **AM1241** | 6.78 | https://pubmed.ncbi.nlm.nih.gov/28045021/ | 7.83 | https://pubmed.ncbi.nlm.nih.gov/23711022/ |
|  |  |  |  |  |
|  | **MEAN** |  | **MEAN** | **SEM** |
|  | **6.78** |  | **7.83** |  |
